# Data on the effect of maternal separation coupled with social isolation in a forced swim test and gene expression of glial fibrillary acid protein in the prefrontal cortex of rats

**DOI:** 10.1016/j.dib.2018.03.055

**Published:** 2018-03-16

**Authors:** Yosuke Yamawaki, Misako Nishida, Kana Harada, Hiroyuki Akagi

**Affiliations:** a Laboratory of Molecular and Cellular Pharmacology, Faculty of Pharmaceutical Sciences, Hiroshima International University, 5-1-1, Hirokoshingai, Kure, Hiroshima 737-0112, Japan; b Laboratory of Neuropharmacology, Faculty of Pharmaceutical Sciences, Hiroshima International University, 5-1-1, Hirokoshingai, Kure, Hiroshima 737-0112, Japan; c Department of Cellular and Molecular Pharmacology, Institute of Biomedical and Health Sciences, Hiroshima University, 1-2-3, Kasumi, Minami-ku, Hiroshima 734-8553, Japan

**Keywords:** GFAP, glial fibrillary acidic protein, MS, maternal separation, qPCR, quantitative polymerase chain reaction, GAPDH, glyceraldehyde-3-phosphate dehydrogenase, PFC, prefrontal cortex, Maternal separation, Early life stress, GFAP, qPCR, Forced swim test

## Abstract

Early life adversity, such as neglect, increases the risk for major depressive disorder and anxiety disorders. It is well-known that astrocytes have key roles in brain function. In this paper, we show the effect of maternal separation (MS) coupled with social isolation on stress response and gene expression of glial fibrillary acidic protein (GFAP) as a marker of astrocytes, in early life and adulthood. Stress response was evaluated by using a forced swim test. GFAP gene expression level was evaluated by using the quantitative polymerase chain reaction (qPCR) method. The data in this article provide indexes affected by early life stress.

**Specifications Table**

**Table:** 

Subject area	*Neuroscience*
More specific subject area	*Neuropsychiatry*
Type of data	*Graph, figure*
How data was acquired	*Immobility time and body weight were manually measured. Gene expression were measured by qPCR using the PikoReal 96 and PikoReal software 2.2 (Thermo Fischer Scientific)*
Data format	*Graphs*
Experimental factors	*Maternal separation (MS) coupled with social isolation of rats from postnatal day 2 (P2) to P14*
Experimental features	*Quantitation of glial fibrillary acid protein (GFAP) gene expression in prefrontal cortex of rats*
Data source location	*Hiroshima, Japan*
Data accessibility	*The data are in this article.*

**Value of the data**

• The effect of maternal separation coupled with social isolation in early life stage lasts until adulthood.
• The data provide a methodology in maternal separation coupled with social isolation to other researchers.
• This data allowed other researchers to easily confirm the establishment of maternal separation coupled with social isolation.

## 1 Data

**Fig. 1:**
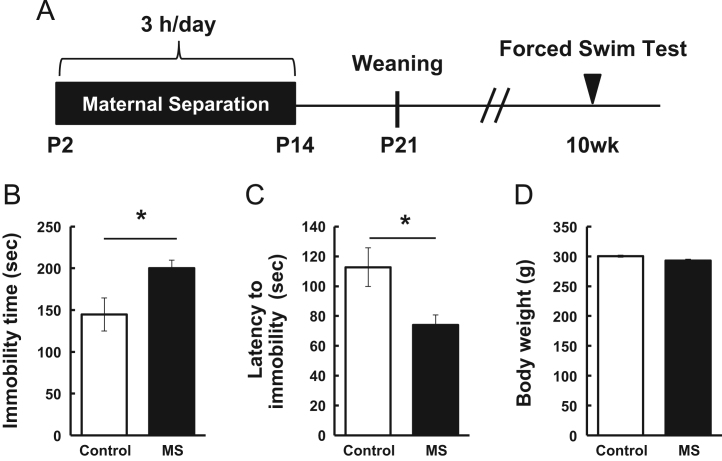
Effect of maternal separation coupled with social isolation in a forced swim test. This figure shows the time schedule of the maternal separation (MS) coupled with social isolation protocol and behavioral tests (A). MS (3 h/day) was performed for 13 consecutive days (from postnatal day 2 (P2) to P14. On day 21, pups were weaned, separated (3 rats/cage), and maintained (B-D). A forced swim test was performed after measuring body weight at age 10 weeks (B, C). Rats were submitted to a forced swim test, and total immobility time (B) and latency to immobility (C) were measured. Body weight was measured before the test (D). Values are presented as means ± S.E.M. * *P* < 0.05 *n* = 12 (Control), *n* = 13 (MS).

**Fig. 2:**
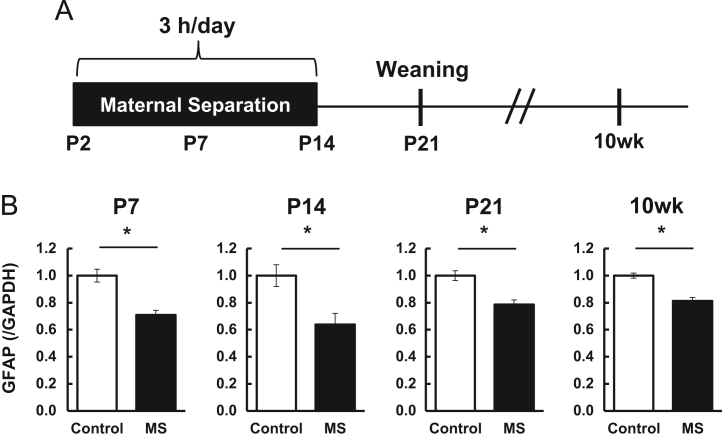
Alteration of glial fibrillary acidic protein mRNA levels in the prefrontal cortex undergone maternal separation coupled with social isolation. Graphs show the glial fibrillary acidic protein (GFAP) mRNA levels in the prefrontal cortex at ages P7, P14, P21, and 10 weeks quantified by quantitative reverse-transcription PCR (qPCR). Values are presented as means ± S.E.M. * *P* < 0.05 [P7; *n* = 10 (Control), *n* = 10 (MS), P14; *n* = 11 (Control), *n* = 11 (MS), P21; *n* = 14 (Control), *n* = 14 (MS), 10 weeks; *n* = 5 (Control), *n* = 6 (MS)].

We evaluated whether or not the maternal separation (MS) coupled with social isolation protocol at the indicated time schedule (Fig. 1A) changed the stress response by using a forced swim test (Fig. 1B-D). As shown in Fig. 2, the levels of gene expression of glial fibrillary acidic protein (GFAP), a marker of astrocytes, in the prefrontal cortex of the rats at 7 days postnatal (P7), P14, P21, and 10 weeks were presented.

## 2 Experimental design, materials and methods

### 2.1 Animals

Ten pregnant female Sprague-Dawley rats were purchased from Charles River Japan (Yokohama, Japan). One hundred and six pups were used in this study. The rats were housed individually in a breeding colony at a constant room temperature of 23 ± 2 °C with a humidity of 60% and a 12 h light/dark cycle with free access to food and water. All experimental procedures were approved by the Animal Research Committee of the Hiroshima International University and performed in accordance with the guidelines of the National Institute of Health.

### 2.2 Maternal separation coupled with social isolation (MS)

The MS coupled with social isolation procedure used in this manuscript has been described previously [1], [2], [3]. The pups were isolated from the dam, nest, and siblings and placed in individual round containers for 3 h per day (9:00 a.m. to 12:00 noon) from postnatal day 2 (P2) to P14. All litters were weaned on P21, separated on the basis of sex, and maintained with *ad libitum* access to food and water. Only male rats were used in behavioral tests.

### 2.3 Forced swim test

Rats were placed into a clear acrylic cylinder (24 cm diameter, 50 cm height), filled to two-thirds of the height with water. The time spent immobile during the 5-min testing period was manually measured by a blinded observer. Immobility was defined as a lack of movement, but included the presence of movement necessary to keep the head above water. The latency to first immobility was scored. Latency was defined as the time from the start of recording to the first state of immobility.

### 2.4 Tissue isolation

The rats were sacrificed by decapitation and their brains were quickly removed at P7, P14, P21, and 10 weeks. The brain was washed with ice-cold saline, and the prefrontal cortex (PFC) was dissected and placed onto an ice-cold plate. These samples were snap-frozen in liquid nitrogen and stored at −80 °C until use.

### 2.5 Quantitative polymerase chain reaction (qPCR)

Tissue samples were homogenized at 10,000 rpm using a Polytron homogenizer and TriPure Isolation Reagent (Roche Diagnostics), and total RNA was isolated according to the manufacturer's protocols. Complementary DNA (cDNA) was synthesized from 0.5 µg of total RNA (final volume 10 µl) using ReverTra Ace qPCR RT Master Mix with gDNA Remover (TOYOBO). Two-step qPCR with THUNDERBIRD SYBR qPCR Mix (TOYOBO) was performed according to the manufacturer's protocol using PikoReal 96 (Thermo Fisher Scientific). GFAP mRNA was used as a marker for astrocytes. The endogenous GAPDH gene was used as an internal control. qPCR was performed using the following primers: GAPDH Forward 5′-TGCCACTCAGAAGACTGTGG-3′, Reverse 5′- TTCAGCTCTGGGATGACCTT-3′; GFAP Forward 5′-GACCGCTTTGCTAGCTACATCG-3′, Reverse 5′-GGTTTCATCTTGGAGCTTCTGC-3′.

### 2.6 Statistics

The data values were presented using the mean ± standard error of the mean (SEM). Statistical analyses were carried out using Student's *t*-test, using JMP 8.0.2 software (SAS, Cary, NC, USA). A *P* value of less than 0.05 was considered statistically significant.
